# Variations in brain DNA

**DOI:** 10.3389/fnagi.2014.00323

**Published:** 2014-11-25

**Authors:** Jesús Avila, Alberto Gómez-Ramos, Eduardo Soriano

**Affiliations:** ^1^Centro de Investigación Biomédica en Red de Enfermedades Neurodegenerativas (CIBERNED), ISCIIIMadrid, Spain; ^2^Centro de Biología Molecular Severo Ochoa (CSIC-UAM), Neurobiology LaboratoryMadrid, Spain; ^3^Department of Cell Biology, Faculty of Biology, University of Barcelona, Developmental Neurobiology and Regeneration Lab, Parc Científic de BarcelonaBarcelona, Spain; ^4^Vall d’Hebrón Institut de Recerca (VHIR)Barcelona, Spain

**Keywords:** SNV, somatic mutations, sequence analysis, DNA, brain diseases, Alzheimer disease

## Abstract

It is assumed that DNA sequences are conserved in the diverse cell types present in a multicellular organism like the human being. Thus, in order to compare the sequences in the genome of DNA from different individuals, nucleic acid is commonly isolated from a single tissue. In this regard, blood cells are widely used for this purpose because of their availability. Thus blood DNA has been used to study genetic familiar diseases that affect other tissues and organs, such as the liver, heart, and brain. While this approach is valid for the identification of familial diseases in which mutations are present in parental germinal cells and, therefore, in all the cells of a given organism, it is not suitable to identify sporadic diseases in which mutations might occur in specific somatic cells. This review addresses somatic DNA variations in different tissues or cells (mainly in the brain) of single individuals and discusses whether the dogma of DNA invariance between cell types is indeed correct. We will also discuss how single nucleotide somatic variations arise, focusing on the presence of specific DNA mutations in the brain.

## Introduction

DNA molecules have been described as the conserved stores of genetic information (Cech, 2012). Whole-genome and exome sequencing are powerful research tools with which to analyze the molecular basis of many human diseases of familial origin (Drmanac, 2012). However, during development or in adulthood, somatic mutations may appear in specific tissues of a human being (or other organism).

With the sequencing data in hand, the bottleneck lies in the bioinformatic analysis (Brunham and Hayden, 2012) required to obtain reliable results (it has been reported that whole-genome sequencing technology has an accuracy of only one false single nucleotide variation per 500 kbp (Roach et al., 2011)).

High-throughput sequencing technologies, like those from Illumina Life Technologies, Ion Torrent, and Roche Diagnostics, are widely used. Other sequence detection methods are based on magnetic tweezers (Linnarsson, 2012) or on other approaches, such as nanopore sequencing analysis, in which single molecules of DNA can be deciphered as they pass through a tiny channel (Pennisi, 2012). All these techniques have facilitated whole-genome or exome sequencing at an unprecedented scale, thus allowing the launch of initiatives such as the 1000 Genomes Project, which seeks to analyze DNA variations in human populations (Clarke et al., 2012). It has been suggested that each genome contains 1.5 10^5^ new single nucleotide variants (SNVs) that are not present in the dbSNP database (Pelak et al., 2010). These variants are present in different genome regions, like the exome, and may be related to genes involved in human diseases (MacArthur et al., 2012).

In this review, we will comment on somatic DNA mutations occurring mainly in the brain.

## How to obtain reliable data in genome (exome) sequencing

Sequence analysis can be done from homogeneous (from a single cell type) or heterogeneous DNA samples. In the first case, Sanger’s method may still be the most accurate analysis to obtain reliable results. However, samples containing heterogeneous (more than five types) DNA molecules cannot be sequenced by this technique, thus requiring alternative methods like Illumina sequencing. However, these massive sequencing techniques can generate artifacts. A preliminary approach to identify these artifacts is through examining the genome database to determine whether an identified variant is already present (thus suggesting that is not an artifact). Another approach, based on the old idea that the DNA sequence in a single organism is identical in every cell of the organism, is to look for possible Mendelian errors. To distinguish false from true variants in the genome of a human being, the sequences of the DNA of his/her parents will indicate the presence of a new variant present in the child but that is absent in the parents. This Mendelian error may suggest a false variant when somatic mutations are not taken into account (Patel et al., 2014). However, this possible false variant may also originate from a somatic mutation occurring specifically in the DNA of the child. This issue can be addressed by obtaining 50–200 reads of each base to confirm the presence of a SNV. Nevertheless, it cannot be concluded that a variant found only at low number of reads is not a true variant. When a heterogeneous sample contains more than one cell type, the difference among the proportion of DNA molecules containing a variant correlates with the proportion of cells in which these molecules are present. If the change in sequence in the somatic cells took place during development, more cells are likely to contain this variation. However, if the change occurred late in adult life, only a small proportion of the cells will hold the DNA modification. It may be difficult to validate such DNA somatic sequences by methods other than those of Illumina. This is a limitation of current next-generation sequencing techniques, which have the sensitivity required for the analysis of heterogeneous DNA samples but generate an (low) error rate.

## Somatic DNA mutations

The development of a human starts from one cell that divides into two, followed by further cell divisions until the organism has generated more than 10^13^ cells in a precisely controlled ontogenetic process (Frank, 2010). During development, failures in DNA replication (or repair) during cell proliferation may occur and result in the appearance of somatic mutations. Afterwards, during adulthood, additional somatic mutations may arise, and genomic variability may encode distinct cell lineages in different tissues (Frumkin et al., 2005). In fact, studies have pointed to the presence of multiple different genomes in a single human being (Lupski, 2013), and differences in DNA sequences have been reported in a variety of tissues (Frank, 2010; Pelak et al., 2010; Clarke et al., 2012; MacArthur et al., 2012; Pennisi, 2012) from the same individual. Depending on the circumstances, the appearance of somatic mutations differs depending on the tissue type. One of these circumstances is the presence of distinct types and amounts of DNA polymerases or repair enzymes in tissues. Low fidelity DNA polymerases can introduce not only nucleotide substitutions but also tandem mutations, as is the case of DNA polymerase ζ (Saribasak et al., 2012). Various forms of mutations may arise, such as sense and non-sense base substitutions (SNP), deletions, and insertions or through other mechanisms like the movement of transportable elements (Lynch, 2010; Vogel, 2011). These variations could change depending on the cell origin. Moreover, some types of cell are more sensitive to DNA damage or changes than others. This is reflected by germinal and somatic cells. The protection of the genome is critical for germ line development. A mechanism to ensure this protection has been described in *Drosophila* (Rangan et al., 2011). This mechanism does not act on somatic cells and consequently this cell type is more susceptible to mutations. However, to facilitate genome maintenance, in response to DNA damaging agents somatic cells have a DNA-damage checkpoint-signaling pathway involving DNA-repair scaffolding proteins like SlX4 (Ohouo et al., 2013). Moreover, specific double breaks are essential for the proper function of specific cells, like sperm cells, because these DNA breaks are required for the correct exchange of DNA (Kauppi et al., 2013).

At the cellular level, considerable efforts have been devoted to minimizing genomic insults in cultured pluripotent stem cells (Weissbein et al., 2014). Additionally, in the whole organism, clonal mosaicism for large chromosomal anomalies, from birth to old age, has been reported (Jacobs et al., 2012; Laurie et al., 2012). At cell cycle level, differences in DNA repair between the phases of the cycle have been described. For instance, during mitosis, DNA double-strand breaks (DSBs) are not repaired. It has been suggested that these mitotic DBSs cause end to end chromosome fusions and that they promote aberrant chromosome segregation (Orthwein et al., 2014).

Also, at the molecular level, not all the bases in the human genome are equally prone to chance mutations (Ponting, 2012). Mutations are more frequently observed in three types of sequences, namely simple repeats, DNAse hypersensitive sites in embryonic stem cells, and some trinucleotide sequences (Michaelson et al., 2012). Also, the expansion of trinucleotide repeats causes some disorders, mainly in neurons and myopathies, which could be caused by a slippage that involves DNA polymerases β and δ (Chan et al., 2013). In contrast, for some cells, like neuroblastoma cells, sensitivity to DNA damage depends on their state of differentiation. Undifferentiated human SH-SY5Y neuroblastoma cells are less sensitive to DNA damage than differentiated cells, in part because they show more efficient base excision repair mechanisms (Sykora et al., 2013).

## Somatic DNA mutation with aging

Some tissues show an increased rate of DNA mutations with aging (Kong et al., 2012). The appearance of somatic mutations, which increases with age, is known to cause or increase susceptibility to diseases like cancer (Moskalev et al., 2012). For example, an age effect on the repair of DNA strand breaks in blood mononuclear cells has been reported (Garm et al., 2013). Aging is also proposed to be a risk factor for neurodegenerative disorders. Indeed, an increasing number of somatic mutations are being associated with neurological diseases (Poduri et al., 2013; Madabhushi et al., 2014; Singleton, 2014).

Furthermore, specific changes in mitochondrial brain DNA have been reported (Bender et al., 2006; Kraytsberg et al., 2006). Mitochondrial DNA (mtDNA) is not protected by histones and is therefore more sensitive to external damage. Indeed, mtDNA deletions are abundant in aged substantia nigra neurons (Bender et al., 2006; Kraytsberg et al., 2006) and in peripheral tissues (Baines et al., 2014). Curiously, mtDNA damage in a mouse model of Alzheimer disease (AD) decreases amyloid beta plaque formation (Pinto et al., 2013). Moreover, DNA ligase activity, which is probably involved in DNA repair, is lower in mitochondrial extracts from AD patients than in matched non-demented controls (Canugovi et al., 2014).

## Mechanisms for DNA sequence variations

Briefly, we will comment on several mechanisms that can give rise to somatic DNA sequence variations. As mentioned, not all the nucleotides in the human genome are equally prone to chance mutations, with exonic sequences and GpG-rich sequences showing greater susceptibility (Michaelson et al., 2012). CpG-rich sequences can also be methylated (or demethylated), and such changes in methylation in neurons affect memory formation (Kaas et al., 2013).

Furthermore, CpG-rich sequences are present in the promoters involved in divergent (on both sides with opposite orientations) transcription. Changes in such sequences may alter gene expression (Wu and Sharp, 2013).

Opening of the double helix facilitates DNA damage. This opening occurs not only during DNA replication, a process in which lesions may occur anywhere in the genome, but also during the active transcription of regions. High levels of transcription induce genomic instability (Wu and Sharp, 2013). A mouse model deficient in DNA repair and transcription shows an increase in DNA damage, which results in premature aging (de Boer et al., 2002). This observation suggests that the opening of the DNA double helix during DNA transcription also facilitates DNA damage, as proposed for replication (Marchesi, 2011). Indeed, during transcription, the coding DNA strand is exposed as a single DNA strand, whereas the non-coding strand is base-paired and protected by the nascent RNA (Aguilera and García-Muse, 2012; Wu and Sharp, 2013). Also, the unwinding of the DNA helix during transcription sometimes generates topological perturbations that can affect genome stability (Bermejo et al., 2012). In addition, somatic hypermutation of immunoglobulin genes has been related to changes in RNA polymerase II progression (Kodgire et al., 2013). Also, it has been described that RNA polymerase and the protein UvrD, a helicase, cooperate to target a damaged DNA site for repair (Epshtein et al., 2014).

## DNA damage repair mechanisms

There are several DNA damage repair mechanisms, these involving base or nucleotide excision repair, mismatch repair, non-homologous end joining or homologous recombination. In base excision repair, it has been proposed that damaged DNA bases are removed through a major pathway involving DNA glycosylases (Caldecott, 2003; Jackson and Bartek, 2009). For example, 7,8-dihydro-8-oxoguanine (8-OHdG), which arises by oxidative damage, is hydrolyzed by MuTYH DNA glycosylases (Markkanen et al., 2013). Also, Nei13 DNA glycosylase may participate in this pathway (Regnell et al., 2012). Recently, the effects of DNA sequence context on the glycosylase activity of human 8-oxoguanine DNA glycosylase have been reported (Sassa et al., 2012).

In addition, the modified base can be identified by a DNA glycosylase and removed by an endonuclease, resulting in a gap in the DNA sequence that is filled by a DNA polymerase and sealed by a DNA ligase (Caldecott, 2003; Maiti et al., 2012). When this repair mechanism is not working properly, the DNA sequence undergoes a mutation (see also Deng et al., 2014) for other repair mechanisms that can result in the introduction of mutations into the genome). The probability of the appearance of a mutation increases in the aged brain when there is an accumulation of 8-OHdG (Wolf et al., 2005). It should be noted that some DNA glycosylases involved in base excision repair contain a Fe-S cluster essential for their activity (Cunningham et al., 1989). Furthermore, oxidative damage specifically in neuronal DNA can also occur. Indeed, the brain has an enhanced cellular metabolism compared to other tissues, and this may result in the formation of free radicals that damage the DNA of brain cells (Hofman, 1991). In this regard, impaired repair of oxidative DNA damage may influence the clinical manifestation of AD (Silva et al., 2014).

Also, the deregulation of the DNA damage response has been described upon intercellular contact in non-neuronal cells (Kang et al., 2012). Nothing is known about whether synaptic neuronal activity facilitates the deregulation of DNA damage. Also, it has been established that ribonucleotides contaminate DNA in some circumstances. Okazaki fragments play an important role in replication. These fragments consist of a short sequence of about 10 ribonucleotides followed by a sequence (about 300 nucleotides) of deoxyribonucleotides. The ribonucleotides must be removed and replaced by deoxyribonucleotides during DNA replication, but if complete removal is impaired, ribonucleotides can be incorporated into nascent DNA. This aberrant incorporation results in DNA damage (Caldecott, 2014), directly or through the formation of DNA single-strand breaks (SSBs), which in turn have been linked to neurodegeneration (Caldecott, 2008). In some conditions, like when the number of rNTPs exceeds that of dNTPs, replicative DNA polymerases incorporate ribonucleotides into DNA (Reijns et al., 2011). This aberrant incorporation can be repaired by enzymatic removal of ribonucleotides from DNA, to preserve genome integrity (Reijns et al., 2012). A mammalian RNAse, RNAse H2, removes ribonucleotides from DNA to maintain genome integrity (Hiller et al., 2012). Also, the structural localization of DNA lesions in nucleosome core particles influences accessibility to base excision repair enzymes (Rodriguez and Smerdon, 2013). In addition, it is postulated that the histone mark H3K36me3 regulates human DNA mismatch repair (Li et al., 2013).

As previously indicated, DNA damage can result in SSBs or DSBs. When occurring in non-dividing cells, such as neurons, these breaks promote premature aging of the cell (Ames et al., 1993), thereby accelerating their demise. The repair of SSBs or DSBs has been extensively reviewed (Ciccia and Elledge, 2010). Single nucleotide excision repair can take place anywhere in the genome (Ciccia and Elledge, 2010), even in actively transcribing regions, as the transcribing RNA polymerase acts as a damage sensor (de Laat et al., 1999; Dogliotti et al., 2001). The result can be the appearance of a mutation. The consequences of mutations will differ depending on whether they take place in introns, upstream sequences, promotors, codifying sequences (exons), etc., since they will affect the regulation of the amount of RNA expressed, RNA stability, and the presence of silent or non-silent mutations in the translated proteins. SSBs can be corrected by various DNA repair mechanisms, and the DNA damage associated with these breaks induces p53 target genes to repair DNA (Smith and Seo, 2002).

DNA DSBs are generated throughout cell life and can result in irreversible damage. To avoid this damage, the cell can generate a new DNA replicate in order to produce homologous templates once again (homology-directed repair) (Jones and Petermann, 2012). In the absence of new DNA replication (a feature of non-dividing cells like neurons), a fast process known as non-homologous end-joining occurs, which repairs DNA breaks by shielding the DNA ends (Sale et al., 2012). In proliferating cells, homology-directed repair is blocked when non-homologous end-joining is active (Lukas and Lukas, 2013). Also, in several neurodegenerative disorders, slippage during replication of repetitive sequences may occur and thus require repair of the newly synthesized strand (Schofield and Hsieh, 2003). These mechanisms are not discussed in this review. However, they can occur in neurons and do not result in the appearance of SNVs but in insertions (or deletions) (indels). Neither will we discuss the regulation of DNA damage responses by ubitiquin and SUMO (Jackson and Durocher, 2013). Moreover, DNA damage can arise by reversal apoptosis in a mechanism that rescues cells from a critical stage named “anastasis” (Tang et al., 2012).

## DNA variations in somatic cells

All the above-mentioned processes related to changes in the DNA sequence of somatic cells may act in a different way in tissues of different origins. Preliminary results from the comparison of DNA sequences of exomes from tissues with an endoderm, mesoderm or exoderm origin support this notion (Gómez-Ramos et al., 2014). Furthermore, somatic mosaicism has been described in human skin (Abyzov et al., 2012).

In addition, in the same tissue, for example the central nervous system, different DNA sequence variations may occur in distinct cell types.

## Variations in brain DNA sequences

Little is known about specific DNA mutations occurring specifically in brain cells. The adult human brain comprises two main cell types, neurons and glia cells, in a roughly 50/50 proportion. The main difference between glia cells and neurons is that the former are proliferating cells that are renewed many times throughout adult life, while the latter It is assumed that DNA sequences are conserved in the diverse cell types present in a multicellular organism like the human being. Thus, in order to compare the sequences in the genome of DNA from different individuals, nucleic acid is commonly isolated from a single tissue. In this regard, blood cells are widely used for this purpose because of their availability. Thus blood DNA has been used to study genetic familiar diseases that affect other tissues and organs, such as the liver, heart, and brain. While this approach is valid for the identification of familial diseases in which mutations are present in parental germinal cells and, therefore, in all the cells of a given organism, it is not suitable to identify sporadic diseases in which mutations might occur in specific somatic cells. This review addresses somatic DNA variations in different tissues or cells (mainly in the brain) of single individuals and discusses whether the dogma of DNA invariance between cell types is indeed correct. We will also discuss how single nucleotide somatic variations arise, focusing on the presence of specific DNA mutations in the brain.are mainly terminally differentiated, non-dividing cells, usually of the same age as the host organism.

**Figure 1:**
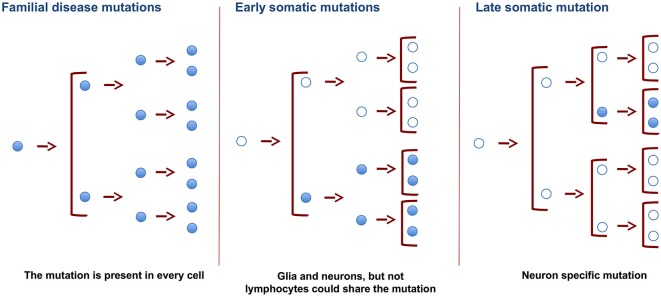
**Changes in DNA of germinal and somatic cells**. SNVs in germinal cells, present in the zygote, will result in the appearance of these SNVs in all the cells of the organism. Changes resulting in SNVs at earlier developmental stages will be present in more cells than those occurring in late adulthood. Late somatic mutations in brain cells may promote specific changes in DNA. These changes may cause the appearance of neurodegenerative disorders.

Some DNA variations (or mutations) can be present in all the cell types of an organism, as is the case of neurodegenerative disorders with a familial origin. In these conditions, germinal cells are responsible for ensuring that the mutations appear in the DNA of every cell of the organism. Also, if the mutation takes place in precursor-dividing cells early in development, both glia and neuron cells carry the same variation in their DNA. However, when it occurs later during development or in adIt is assumed that DNA sequences are conserved in the diverse cell types present in a multicellular organism like the human being. Thus, in order to compare the sequences in the genome of DNA from different individuals, nucleic acid is commonly isolated from a single tissue. In this regard, blood cells are widely used for this purpose because of their availability. Thus blood DNA has been used to study genetic familiar diseases that affect other tissues and organs, such as the liver, heart, and brain. While this approach is valid for the identification of familial diseases in which mutations are present in parental germinal cells and, therefore, in all the cells of a given organism, it is not suitable to identify sporadic diseases in which mutations might occur in specific somatic cells. This review addresses somatic DNA variations in different tissues or cells (mainly in the brain) of single individuals and discusses whether the dogma of DNA invariance between cell types is indeed correct. We will also discuss how single nucleotide somatic variations arise, focusing on the presence of specific DNA mutations in the brain.ulthood, this variation may be specific for a cell type or lineage (Figure 1). Also, in adult cells, somatic DNA variations may be specific for a cell type, and the mechanism for these variations may differ in glia and neurons.

## Limitations to detect SNVs in neuronal populations

The presence of various cell types in a single tissue hinders the detection of somatic cell variations using some techniques (with a low sensitivity), like Sanger’s sequencing. Regarding the presence of a DNA mutation in specific cells within brain tissue, when a mutation is present exclusively in neurons, a maximum of only 50% of the cells will show the DNA change; however, when the variation occurs in a specific neuronal population, this percentage is much lower. This scenario impedes reliable results because dye-terminator sequencing (with a higher sensitivity) data is usually validated by Sanger’s method (with a lower sensitivity), which is not suitable for these types of sample. Moreover, dye-terminator sequencing can generate errors because of low signal to noise ratios and non- or mis-detection of the fluorescent signal (Breslow et al., 2008). However, the problem can be partially solved by increasing the number of reads of the DNA regions containing the variations during dye-terminator sequencing. Also, quality filtering techniques may improve Illumina sequencing results (Bokulich et al., 2013).

One of the major difficulties of making such analysis in living human brains is that the taking of human samples from brains must not be done because is a very invasive method. Thus, samples should be taken from autopsies. On the other hand there is a high cellular complexity (many cell types). Recently reported methods of single-cell sequencing are promising for the detection of individual variations in a single cell but they are not fully developed, and the extensive PCR-based amplification used in this method might interfere with the resolution of this approach (Eberwine et al., 2014).

## Brain cells and DNA sequences

During DNA replication in glia cells, erroneous insertions of a base in the newly synthesized strand, which does not match the parental strand, can generate a mutation if the mismatch is not repaired. This type of mutation does not arise in the case of non-proliferating cells like neurons. In neurons (and also in glia cells), changes in the DNA sequence can be associated with other forms of DNA damage. Damage promoted by the extracellular environment can be solved, or not, depending on the DNA repair mechanism present in each cell type. These repair mechanisms involve various proteins, polymerases, ligases, nucleases, and helicases. Also, small RNAs have been implicated in DNA repair (Wei et al., 2012), and other factors like ubiquitin and SUMO may also participate in this process (Ulrich, 2012), as previously described. Histone ubiquitylation, a process regulated by various E3 ligases, is a main response to DSBs (Gudjonsson et al., 2012). The amount of messenger RNAs expressing these repair proteins in brain cell types differs (see Allen Brain Atlas (Hawrylycz et al., 2012)). Thus these differences, together with other distinct transcription levels in different neuronal types, can result in damage to specific neurons, which may lead to the onset of neurodegeneration. Indeed, our preliminary data indicate the presence of a number of SNVs in hippocampal neurons that are absent in cerebellar neurons (Parcerisas et al., 2014). The DNA damage response may involve many molecular changes to ensure correct DNA repair. Some of these changes have been studied in depth (Beli et al., 2012).

## Aging and oxidative damage to DNA

Aging is a major risk for DNA damage. Aging can bring about an increase in neuronal DNA lesions (Sedelnikova et al., 2004), which activate the Ras signaling pathway (Boldogh et al., 2012). In neurons, it may result in defects in dendritic spine development and synapse formation (Yang et al., 2013). The damage can be due, at least in part, to the high levels of reactive oxygen species (ROS) and the low levels of anti-oxidant defenses in these brain cells (Finkel and Holbrook, 2000).

ROS-induced DNA damage can lead to the formation of 8-OHdG (see below).

Also, it has been reported that post-mitotic neurons develop a p21-dependent senescence phenotype driven by a DNA damage response (Jurk et al., 2012). On the other hand, it has been found that DNA methylation declines with age (Heyn et al., 2012). However, in this short review, we will not address changes in the epigenome but only briefly comment that chromatin actively participates in the DNA damage response (Soria et al., 2012). Although DNA replication takes place in S phase, the DNA damage response occurs at any point of the cell cycle and in differentiated, non-proliferating cells like neurons. This response occurs in the context of chromatin in eukaryotic cells, where DNA is wrapped with histone proteins, and there are some variations in proteins, like H2A-X, whose phosphorylation is increased in the DNA damage response (Hiller et al., 2012).

The DNA damage response is characterized by the activation of a kinase such as H2A-X (as previously indicated), which modifies histones, or by the induction of proteins like p53 or p21, which contribute to cell cycle arrest. Also, the acetylation of some proteins is involved in this response (Robert et al., 2011), and specific acetylation of p53 by HDAC inhibition prevents DNA damage in neurons (Brochier et al., 2013). Furthermore, DNA damage, mainly in the form of DSBs, may lead to cellular senescence.

Regarding new neuronal cells, adult neurogenesis requires a stringent genomic maintenance program to ensure the correct transmission of the genetic program to newborn neurons. A factor in this program is TopBP1, a protein linked to DNA replication that is essential for the maintenance of genome integrity during the proliferation of neuronal precursors. A failure in this protein may promote a modification of DNA in the affected neuronal precursors (Lee et al., 2012). Also, regarding transposons, it has been reported that some small RNAs trigger the formation of a class of small RNAs that silence transposon targets (Xiol and Pillai, 2012).

More recently, it has been described that brain activity causes DNA DSBs in neurons and that these breaks are exacerbated by the presence of amyloid beta (Suberbielle et al., 2013). It is not clear yet whether this exacerbation is due to an increase in the transcription of specific genes.

## Mechanisms for DNA sequence variations in brain

Neuron DNA damage, resulting in the appearance of SNVs, can favor neurodegeneration and cognitive decline in diseases like AD (Brasnjevic et al., 2008; Moreira et al., 2008; Suberbielle et al., 2013).

**Figure 2:**
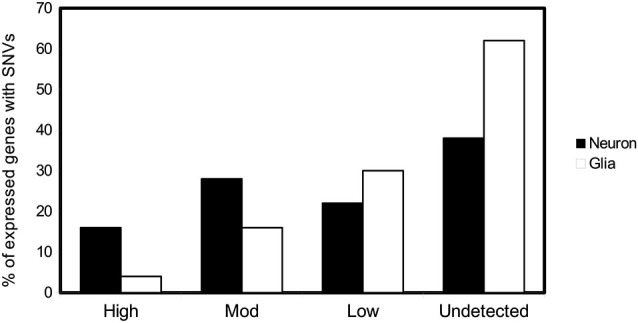
**SNVs in neuron and glia**. Possible relationship with DNA transcription or DNA replication. Percentage of genes with at least one SNV expressed in hippocampal neuron and glial cells (Parcerisas et al., 2014) that are translated into proteins according to the expression levels provided by the database *The Human Protein Atlas*.^1^ The levels of expression of the first 200 genes containing the most hippocampal-specific SNVs (according to a Fisher test respect to the SNVs found in blood), as shown by immunological detection, were checked one by one in the database and their expression in this tissue was annotated.

These SNVs are present after DNA opening, DNA damage, and/or inefficient repair. Opening of the DNA double helix occurs by two mechanisms, namely DNA replication and DNA transcription. Various proteins, present in different amounts, related to DNA replication transcription or reparation are expressed in neurons and glia cells, as clearly indicated in the Allen Brain Atlas (Hawrylycz et al., 2012). For neurons, the opening of the helix takes place during transcription. Gene expression in the adult mammalian brain is complex. It has been suggested that at least 80% of all genes are expressed in the central nervous system (Lein et al., 2007; Hawrylycz et al., 2012). For glia, DNA opening occurs during both DNA replication and transcription. It has been hypothesized that transcription may be involved mainly in the appearance of DNA variations in neuronal cells, late in development or in the adult organism. We have been examining the SNVs of 200 genes in hippocampal tissue (see legend of Figure 2). Based on the data of the Human Protein Atlas and on the basis of the transcription/translation level of these genes, we have divided them into high, moderate, low, or undetected expression. Thus, we have tested the percentage of SNVs in genes expressed at different levels in neurons and glia cells. We found a higher percentage of DNA changes in cells with high expression of neuronal genes than those with low expression (Figure 2). These observations point to a greater relationship between transcription level and DNA damage in neuronal cells, while DNA damage in glia cells is caused mainly by DNA replication.

Regarding a possible relation between DNA transcription and DNA damage, a ubiquitin-driven link between gene expression and the DNA damage response has been put forward (Shiloh et al., 2011). On the other hand, the presence of DNA loops during transcription may facilitate oxidation, mainly of deoxyguanosine, by ROS (Cadet et al., 1997), yielding the formation of 8-OHdG. Indeed, it has been proposed that oxidative DNA damage initiates neurodegenerative diseases (Perry et al., 2001). In this regard, the damage is not exerted only at the DNA molecule, but unassembled DNA bases can also be oxidized, and these modified bases can be misincorporated into DNA (Luo et al., 2010). However, there is a mechanism—through the enzyme MTH1—that hydrolyzes oxidized DNA bases, thus preventing misincorporation (Dominissini and He, 2014).

## Variations in neuron DNA amount and sequences and their possible association with neurological diseases

Although most neurons are diploid, a small population of these cells surpasses the diploid level (Fischer et al., 2012). These hyperploid neurons are selectively affected by cell death at early stages of AD (Arendt et al., 2010). More recently, an increase in X chromosome aneuploidy has been reported in brain cells of female AD patients (Sugiyama et al., 2014).

By testing DNA from hippocampus, researchers recently revealed the presence of SNVs or insertions/deletions (indels) in AD patients but not in demented controls (Parcerisas et al., 2014). Little is known about the mechanism underlying indels, although a process similar to that of RNA-guided human genome engineering via cas9 has been proposed (Cho et al., 2013; Mali et al., 2013). However, whether this type of mechanism takes place in aging or in AD remains to be elucidated. Finally, transposons are found in brain DNA (Singer et al., 2010; Vogel, 2011), although no association has been made with AD. In addition, aging and AD are characterized by a decreased capacity for DNA repair as a result of a reduction in DNA end-joining activity (a process that calls for DNA-dependent protein kinase activity) required to repair DSBs (Kanungo, 2013).

## Variations in DNA sequences of brain cells obtained from AD patients

The most predominant neurodegenerative disorder is AD (Selkoe, 2011). This disease has been divided into two types, the familial type of predominantly early onset, and the sporadic type, with no familial association, of later onset (Bertram et al., 2010). For familial cases (FAD), genome sequence analysis of DNA from the patients’ lymphocytes have indicated that the disease is caused by mutations in three genes (*APP*, *PS-1* and *PS-2*) (Selkoe, 2011). To look for possible genetic risk factors in sporadic AD (SAD), genome-wide association studies (GWAS) have been carried out in patients with this disease (Manolio et al., 2009; Lambert et al., 2013; Ridge et al., 2013; Zhang et al., 2013), also using DNA from lymphocytes. Also, a higher frequency of DNA damage in blood lymphocytes of SAD patients compared with age-matched controls has been reported (Zivković et al., 2013). This observation could be attributable to the presence of a different DNA damage repair mechanism (Leandro et al., 2013).

Early work revealed that the ε4 allele of *APOE* is a strong risk factor for AD. Other risk factors include the presence of SNVs as specific sequences in genes like: *ABCS7, BIN1, CD33, CD2AP, CLU, CR1, EPHA1, MS4A4E/MS4A6A, PICALM* and* SORL1* (Schellenberg and Montine, 2012). In addition, two new susceptibility genes for AD have been reported (Escott-Price et al., 2014), and recently an AD-associated polymorphism in human OGG1 that sensitizes cells to DNA damage has been described (Jacob et al., 2013).

However, a significant proportion of the possible genetic defects related to the development of SAD remains unexplained. This missing defect has been named the “dark matter” of GWAS (Manolio et al., 2009), in an attempt to explain the “missing heritability” by means of GWAS analysis using DNA from lymphocytes (Manolio et al., 2009). Furthermore, little has been reported about the presence of a specific type of mutation, through insertions or deletions (indels), in AD.

Our hypothesis is that some genetic defects of SAD are present only in somatic mutations in neurons but not in peripheral cells like lymphocytes or in the germ line, as is the case for FAD patients (Figure 1). These somatic defects, which are specific to neuronal tissue, are postulated to favor the appearance of late onset dementia in SAD patients.

Preliminary data have indicated the presence of specific mutations in brain tissue from AD patients that are not present in the blood of these patients (Parcerisas et al., 2014). The proteins expressed by these brain genes include transcription factors, ion channels, and proteins related to lipid transport and metabolism, to the cytoskeleton, etc… The possible relation between variations in these genes and neurological disorders deserves further attention. Although mainly non-silent exome DNA variations have been characterized, the analysis of silent exome DNA SNVs could also be of interest due to the consequences of the codon bias in gene expression (Plotkin and Kudla, 2011).

## Role of beta amyloid and tau protein in DNA damage

About a century ago, Alzheimer described the presence of two aberrant structures, senile plaques and neurofibrillary tangles, in the brain of a demented patient (Alzheimer, 1907). We now know that the main component of the plaques is beta amyloid peptide and that of tangles is tau protein.

It has been proposed that Aβ peptide exerts DNA nicking activity that promotes DNA damage and that such damage may occur in AD patients (Gupta et al., 2013). Brain activity causes DNA DSBs in neurons, and these breaks are exacerbated by amyloid-β (Suberbielle et al., 2013). It has recently been shown that NAD attenuates oxidative DNA damage induced by Aβ peptide in cultured cortical neurons (Wu et al., 2014).

Furthermore, mutations in the *MAPT* gene cause chromosome instability and can introduce copy number variation in the genome (Rossi et al., 2013) (see also ref Rossi et al., 2008). Tau protein is not only a cytoskeletal protein but it is found in the nucleus of neurons (Wang et al., 1993; Brady et al., 1995; Lambert et al., 1995; Sultan et al., 2011). Indeed, tau is a DNA-binding protein (Corces et al., 1980; Krylova et al., 2005; Wei et al., 2008; Camero et al., 2014) and it plays a major role in neuronal DNA protection (Violet et al., 2014). Also it has been described that a high level of cytoplasm tau protein promotes neurodegeneration via DNA damage, heterochromatin relaxation, and piwi-like RNA-mediated gene silencing 1 (*PIWIL-1*), thus facilitating cell cycle re-entry (Frost et al., 2014). The differences between the two effects exerted by tau can be explained by its form and distribution. Dephosphorylated tau localizes in the nucleus, playing a protective role. In contrast, phosphorylated tau, present in the cytoplasm, interacts with mitochondrial protein *DRP1*, impairing mitochondrial function and facilitating the production of ROS and DNA damage (DuBoff et al., 2012; Manczak and Reddy, 2012; Camero et al., 2014).

In summary, brain-specific DNA changes occur. Thus brain DNA rather than blood DNA should be used to analyze somatic DNA variations linked to neurological diseases and present in brain cells.

## Conflict of interest statement

The authors declare that the research was conducted in the absence of any commercial or financial relationships that could be construed as a potential conflict of interest.

## Footnotes

^1^http://www.proteinatlas.org/

## Glossary

*De novo mutation*: new mutation. Alteration in a gene that appears for the first time in a member of a family as a result of a mutation produced in a germ cell (egg or sperm) or in the fertilized egg. *Germinal mutation*: mutation that occurs in a germ cell (egg or sperm) and can be transmitted to the offspring. *Somatic mutation*: mutation that is not inherited from parents and not transferred to the offspring. They can occur in any of the cells except the germ cells. *Exome*: part of the genome composed by the exons, the portions of the genes containing the information to synthesize the proteins. *Mosaicism*: it describes a condition in which one individual who has developed from a single fertilized egg, has two or more populations of cells with different genotypes. If these different populations of cells are in a somatic tissue, we talk about **somatic mosaicism**. We refer to **germline mosaicism** when some gametes (oocytes or sperm) carry a mutation in an individual cell, but the rest of cells are normal.
